# Supplementary material for: Perceptions of 3R implementation in European animal research: A systematic review, meta-analysis, and meta-synthesis of barriers and facilitators
Source: PLoS One. 2024 Mar 28;19(3):e0300031. doi: 10.1371/journal.pone.0300031 (PMC10977722; doi:10.1371/journal.pone.0300031)
Supplement: S2 Appendix — (DOCX) [file pone.0300031.s002.docx]

**S2 Appendix. Search strategy used for the systematic review.**

**PubMed**

("3R"[Title/Abstract] OR "3Rs"[Title/Abstract] OR "Three R"[Title/Abstract] OR "Three Rs"[Title/Abstract] OR ("reduc*"[Title/Abstract] AND "replac*"[Title/Abstract] AND "refin*"[Title/Abstract]) OR "new approach method*"[Title/Abstract] OR "non animal model*"[Title/Abstract] OR "non animal method*"[Title/Abstract] OR "non-animal model*"[Title/Abstract] OR "non-animal method*"[Title/Abstract] OR "NAM"[Title/Abstract] OR "NAMs"[Title/Abstract] OR (("animal use"[Title/Abstract] OR "animal testing"[Title/Abstract]) AND ("alternative*"[Title/Abstract] OR "substitut*"[Title/Abstract])) OR "Animal Welfare"[Title/Abstract] OR "Animal Experimentation"[Title/Abstract] OR "Animal Welfare"[MeSH Terms] OR "Animal Experimentation"[MeSH Terms]) AND ("scientist*"[Title/Abstract] OR "animal welfare officer*"[Title/Abstract] OR "researcher*"[Title/Abstract] OR "student*"[Title/Abstract] OR "investigator*"[Title/Abstract] OR "veterinar*"[Title/Abstract] OR "expert*"[Title/Abstract] OR "committee*"[Title/Abstract] OR "animal techn*"[Title/Abstract] OR "animal caregiver*"[Title/Abstract] OR "Laboratory Personnel"[Title/Abstract] OR "Medical Laboratory Personnel"[MeSH Terms] OR "Veterinarians"[MeSH Terms] OR "Laboratory Personnel"[MeSH Terms] OR "Research Personnel"[MeSH Terms] OR "Students"[MeSH Terms]) AND ("austria*"[Title/Abstract] OR "belgi*"[Title/Abstract] OR "France"[Title/Abstract] OR "French"[Title/Abstract] OR "Paris"[Title/Abstract] OR "german*"[Title/Abstract] OR "Berlin"[Title/Abstract] OR "Czech"[Title/Abstract] OR "Ireland"[Title/Abstract] OR "Irish"[Title/Abstract] OR "ital*"[Title/Abstract] OR "Rome"[Title/Abstract] OR "Sicily"[Title/Abstract] OR "luxembourg*"[Title/Abstract] OR "Netherlands"[Title/Abstract] OR "Holland"[Title/Abstract] OR "Dutch"[Title/Abstract] OR "norw*"[Title/Abstract] OR "Svalbard"[Title/Abstract] OR "Poland"[Title/Abstract] OR "Polish"[Title/Abstract] OR "portug*"[Title/Abstract] OR "romania*"[Title/Abstract] OR "slovak*"[Title/Abstract] OR "Spain"[Title/Abstract] OR "Spanish"[Title/Abstract] OR "swed*"[Title/Abstract] OR "Switzerland"[Title/Abstract] OR "Swiss"[Title/Abstract] OR "United Kingdom"[Title/Abstract] OR "UK"[Title/Abstract] OR "brit*"[Title/Abstract] OR "engl*"[Title/Abstract] OR "London"[Title/Abstract] OR "scot*"[Title/Abstract] OR "Hebrides"[Title/Abstract] OR "Wales"[Title/Abstract] OR "Welsh"[Title/Abstract] OR "Channel Islands"[Title/Abstract] OR "Guernsey"[Title/Abstract] OR "Denmark"[Title/Abstract] OR "Greenland"[Title/Abstract] OR "Danish"[Title/Abstract] OR "ukrain*"[Title/Abstract] OR "Austria"[MeSH Terms] OR "Belgium"[MeSH Terms] OR "France"[MeSH Terms] OR "Germany"[MeSH Terms] OR "Czech Republic"[MeSH Terms] OR "Ireland"[MeSH Terms] OR "Italy"[MeSH Terms] OR "Luxembourg"[MeSH Terms] OR "Netherlands"[MeSH Terms] OR "Norway"[MeSH Terms] OR "Poland"[MeSH Terms] OR "Portugal"[MeSH Terms] OR "Romania"[MeSH Terms] OR "Slovakia"[MeSH Terms] OR "Spain"[MeSH Terms] OR "Sweden"[MeSH Terms] OR "Switzerland"[MeSH Terms] OR "United Kingdom"[MeSH Terms] OR "Denmark"[MeSH Terms] OR "Ukraine"[MeSH Terms]) AND 2013/01/01:2023/01/01[Date - Publication]

**Web of Science (all collections, all databases)**

((TI=3R OR AB=3R) OR (TI=3Rs OR AB=3Rs) OR (TI="Three R" OR AB="Three R") OR (TI="Three Rs" OR AB="Three Rs") OR ((TI=reduc* OR AB=reduc*) AND (TI=replac* OR AB=replac*) AND (TI=refin* OR AB=refin*)) OR (TI="new approach method*" OR AB="new approach method*") OR (TI="non animal model*" OR AB="non animal model*") OR (TI="non animal method*" OR AB="non animal method*") OR (TI="non-animal model*" OR AB="non-animal model*") OR (TI="non-animal method*" OR AB="non-animal method*") OR (TI=NAM OR AB=NAM) OR (TI=NAMs OR AB=NAMs) OR (((TI="animal use" OR AB="animal use") OR (TI="animal testing" OR AB="animal testing")) AND ((TI=alternative* OR AB=alternative*) OR (TI=substitut* OR AB=substitut*))) OR (TI="Animal Welfare" OR AB="Animal Welfare") OR (TI="Animal Experimentation" OR AB="Animal Experimentation")) AND ((TI=scientist* OR AB=scientist*) OR (TI="animal welfare officer*" OR AB="animal welfare officer*") OR (TI=researcher* OR AB=researcher*) OR (TI=student* OR AB=student*) OR (TI=investigator* OR AB=investigator*) OR (TI=veterinar* OR AB=veterinar*) OR (TI=expert* OR AB=expert*) OR (TI=committee* OR AB=committee*) OR (TI="animal techn*" OR AB="animal techn*") OR (TI="animal caregiver*" OR AB="animal caregiver*") OR (TI="Laboratory Personnel" OR AB="Laboratory Personnel")) AND ((TI=austria* OR AB=austria*) OR (TI=belgi* OR AB=belgi*) OR (TI=France OR AB=France) OR (TI=French OR AB=French) OR (TI=Paris OR AB=Paris) OR (TI=german* OR AB=german*) OR (TI=Berlin OR AB=Berlin) OR (TI=Czech OR AB=Czech) OR (TI=Ireland OR AB=Ireland) OR (TI=Irish OR AB=Irish) OR (TI=ital* OR AB=ital*) OR (TI=Rome OR AB=Rome) OR (TI=Sicily OR AB=Sicily) OR (TI=luxembourg* OR AB=luxembourg*) OR (TI=Netherlands OR AB=Netherlands) OR (TI=Holland OR AB=Holland) OR (TI=Dutch OR AB=Dutch) OR (TI=norw* OR AB=norw*) OR (TI=Svalbard OR AB=Svalbard) OR (TI=Poland OR AB=Poland) OR (TI=Polish OR AB=Polish) OR (TI=portug* OR AB=portug*) OR (TI=romania* OR AB=romania*) OR (TI=slovak* OR AB=slovak*) OR (TI=Spain OR AB=Spain) OR (TI=Spanish OR AB=Spanish) OR (TI=swed* OR AB=swed*) OR (TI=Switzerland OR AB=Switzerland) OR (TI=Swiss OR AB=Swiss) OR (TI="United Kingdom" OR AB="United Kingdom") OR (TI=UK OR AB=UK) OR (TI=brit* OR AB=brit*) OR (TI=engl* OR AB=engl*) OR (TI=London OR AB=London) OR (TI=scot* OR AB=scot*) OR (TI=Hebrides OR AB=Hebrides) OR (TI=Wales OR AB=Wales) OR (TI=Welsh OR AB=Welsh) OR (TI="Channel Islands" OR AB="Channel Islands") OR (TI=Guernsey OR AB=Guernsey) OR (TI=Denmark OR AB=Denmark) OR (TI=Greenland OR AB=Greenland) OR (TI=Danish OR AB=Danish) OR (TI=ukrain* OR AB=ukrain*)) AND DOP=2013-01-01/2023-01-01

**Scopus (advanced search)**

(TITLE-ABS(3R) OR TITLE-ABS(3Rs) OR TITLE-ABS("Three R") OR TITLE-ABS("Three Rs") OR (TITLE-ABS(reduc*) AND TITLE-ABS(replac*) AND TITLE-ABS(refin*)) OR TITLE-ABS("new approach method*") OR TITLE-ABS("non animal model*") OR TITLE-ABS("non animal method*") OR TITLE-ABS("non-animal model*") OR TITLE-ABS("non-animal method*") OR TITLE-ABS(NAM) OR TITLE-ABS(NAMs) OR ((TITLE-ABS("animal use") OR TITLE-ABS("animal testing")) AND (TITLE-ABS(alternative*) OR TITLE-ABS(substitut*))) OR TITLE-ABS("Animal Welfare") OR TITLE-ABS("Animal Experimentation") OR INDEXTERMS("Animal Welfare") OR INDEXTERMS("Animal Experimentation")) AND (TITLE-ABS(scientist*) OR TITLE-ABS("animal welfare officer*") OR TITLE-ABS(researcher*) OR TITLE-ABS(student*) OR TITLE-ABS(investigator*) OR TITLE-ABS(veterinar*) OR TITLE-ABS(expert*) OR TITLE-ABS(committee*) OR TITLE-ABS("animal techn*") OR TITLE-ABS("animal caregiver*") OR TITLE-ABS("Laboratory Personnel") OR INDEXTERMS("Medical Laboratory Personnel") OR INDEXTERMS(Veterinarians) OR INDEXTERMS("Laboratory Personnel") OR INDEXTERMS("Research Personnel") OR INDEXTERMS(Students)) AND (TITLE-ABS(austria*) OR TITLE-ABS(belgi*) OR TITLE-ABS(France) OR TITLE-ABS(French) OR TITLE-ABS(Paris) OR TITLE-ABS(german*) OR TITLE-ABS(Berlin) OR TITLE-ABS(Czech) OR TITLE-ABS(Ireland) OR TITLE-ABS(Irish) OR TITLE-ABS(ital*) OR TITLE-ABS(Rome) OR TITLE-ABS(Sicily) OR TITLE-ABS(luxembourg*) OR TITLE-ABS(Netherlands) OR TITLE-ABS(Holland) OR TITLE-ABS(Dutch) OR TITLE-ABS(norw*) OR TITLE-ABS(Svalbard) OR TITLE-ABS(Poland) OR TITLE-ABS(Polish) OR TITLE-ABS(portug*) OR TITLE-ABS(romania*) OR TITLE-ABS(slovak*) OR TITLE-ABS(Spain) OR TITLE-ABS(Spanish) OR TITLE-ABS(swed*) OR TITLE-ABS(Switzerland) OR TITLE-ABS(Swiss) OR TITLE-ABS("United Kingdom") OR TITLE-ABS(UK) OR TITLE-ABS(brit*) OR TITLE-ABS(engl*) OR TITLE-ABS(London) OR TITLE-ABS(scot*) OR TITLE-ABS(Hebrides) OR TITLE-ABS(Wales) OR TITLE-ABS(Welsh) OR TITLE-ABS("Channel Islands") OR TITLE-ABS(Guernsey) OR TITLE-ABS(Denmark) OR TITLE-ABS(Greenland) OR TITLE-ABS(Danish) OR TITLE-ABS(ukrain*) OR INDEXTERMS(Austria) OR INDEXTERMS(Belgium) OR INDEXTERMS(France) OR INDEXTERMS(Germany) OR INDEXTERMS("Czech Republic") OR INDEXTERMS(Ireland) OR INDEXTERMS(Italy) OR INDEXTERMS(Luxembourg) OR INDEXTERMS(Netherlands) OR INDEXTERMS(Norway) OR INDEXTERMS(Poland) OR INDEXTERMS(Portugal) OR INDEXTERMS(Romania) OR INDEXTERMS(Slovakia) OR INDEXTERMS(Spain) OR INDEXTERMS(Sweden) OR INDEXTERMS(Switzerland) OR INDEXTERMS("United Kingdom") OR INDEXTERMS(Denmark) OR INDEXTERMS(Ukraine)) AND (LIMIT-TO (PUBYEAR,2022) OR LIMIT-TO (PUBYEAR,2021) OR LIMIT-TO (PUBYEAR,2020) OR LIMIT-TO (PUBYEAR,2019) OR LIMIT-TO (PUBYEAR,2018) OR LIMIT-TO (PUBYEAR,2017) OR LIMIT-TO (PUBYEAR,2016) OR LIMIT-TO (PUBYEAR,2015) OR LIMIT-TO (PUBYEAR,2014) OR LIMIT-TO (PUBYEAR,2013))
