# Supplementary material for: Perceptions of 3R implementation in European animal research: A systematic review, meta-analysis, and meta-synthesis of barriers and facilitators
Source: PLoS One. 2024 Mar 28;19(3):e0300031. doi: 10.1371/journal.pone.0300031 (PMC10977722; doi:10.1371/journal.pone.0300031)
Supplement: S3 Appendix — (DOCX) [file pone.0300031.s003.docx]

**S3 Appendix. R program used for proportion meta-analysis.**

*# Coding UTF-8*

*# R version 4.2.1*

***###################################################################***

***########################### INITIALISATION #########################***

***##################################################################***

*# Load basic packages*

library(readr)

library(dplyr)

library(stringr)

*# Import File to be read*

File <- read_csv("XXXX.csv")

***###################################################################***

***################# TRANSFORMATION OF PROPORTIONS ###############***

***##################################################################***

*# Logit transformation for proportions [1]*

*# When p=0 or p=1, a small increment is added in order to have a finite result [2]*

File <- mutate(File, Logit = case_when(

(Proportion_Agreement == 1) | (Proportion_Agreement == 0) ~ log((Proportion_Agreement+(1/(2*Sample_size))/(1-Proportion_Agreement+(1/(2*Sample_size))))),

TRUE ~ log(Proportion_Agreement/(1-Proportion_Agreement))

)

)

*# Approximate variance of the transformed proportion [1]*

*# When p=0 or p=1, a small increment is added in order to have a finite result [2]*

File <- mutate(File, Variance = case_when(

(Proportion_Agreement == 1) | (Proportion_Agreement == 0) ~ (1/(Sample_size*Proportion_Agreement+0.5))+(1/(Sample_size*(1-Proportion_Agreement)+0.5)),

TRUE ~ (1/(Sample_size*Proportion_Agreement))+(1/(Sample_size*(1-Proportion_Agreement)))

)

)

*# Asymptotic 95% confidence interval for individual studies [1]*

*# For studies with a small sample size (N <= 30), the Wilson score interval is calculated, as it is a better approximation [3]*

File <- mutate(File, CI_95_inf = case_when(

Sample_size <= 30 ~ round((2*Sample_size*Proportion_Agreement+(1.96^2)-1.96*sqrt((1.96^2)+4*Sample_size*Proportion_Agreement*(1-Proportion_Agreement)))/(2*(Sample_size+(1.96^2))), digits = 3),

TRUE ~ round(1/(1+exp(-(Logit-1.96*sqrt(Variance)))), digits = 3) #Include back-transformation of logit transformation

)

)

File <- mutate(File, CI_95_sup = case_when(

Sample_size <= 30 ~ round((2*Sample_size*Proportion_Agreement+(1.96^2)+1.96*sqrt((1.96^2)+4*Sample_size*Proportion_Agreement*(1-Proportion_Agreement)))/(2*(Sample_size+(1.96^2))), digits = 3),

TRUE ~ round(1/(1+exp(-(Logit+1.96*sqrt(Variance)))), digits = 3) #Include back-transformation of logit transformation

)

)

***###################################################################***

***## INVERSE VARIANCE META-ANALYSIS WITH RANDOM EFFECT MODEL ##***

***##################################################################***

yi <- File$Logit

vi <- File$Variance

wi <- 1/vi

*# Paule-Mandel tau^2 estimator through iterative DerSimonian and Laird method (adapted from [4])*

yw <- sum(wi*yi)/sum(wi) *#Fixed-effect estimate*

est_tau2 <- (sum(wi*((yi-yw)^2))-(sum(wi*vi)-(sum((wi^2)*vi)/sum(wi))))/(sum(wi)-(sum(wi^2)/sum(wi)))

est_tau2 <- ifelse(est_tau2 < 0, 0, est_tau2) *#Initial estimation of tau^2*

tau2_DL <- 100

tau2_PM <- est_tau2

b <- 0

while (abs(tau2_DL-tau2_PM) > 0.0001)

{ *#Stay in loop until the difference between two consecutive estimates is smaller than 0.0001*

b <- b+1 *#Add 1 to counter*

tau2_DL <- tau2_PM *#Update previous estimation*

ai <- 1/(vi+tau2_DL) *#Update weights*

yw <- sum(ai*yi)/sum(ai) *#Update Fixed-effect estimate*

tau2_PM <- (sum(ai*((yi-yw)^2))-(sum(ai*vi)-(sum((ai^2)*vi)/sum(ai))))/(sum(ai)-(sum(ai^2)/sum(ai)))

tau2_PM <- ifelse(tau2_PM < 0, 0, tau2_PM) *#Update new estimation*

if (b == 100)

{ *#Break out loop if more than 100 iterations are needed and return the initial estimation*

tau2_PM <- est_tau2

break

}

}

*# Summary effect error variance & Relative weight of studies [5]*

wi <- 1/(vi+tau2_PM)

Meta_Variance <- 1/sum(wi)

File <- mutate(File, Relative_weight = round(Meta_Variance/(Variance+tau2_PM), digits = 3))

*# Summary effect estimation [1]*

Pooled_logit <- sum(wi*yi)/sum(wi)

Meta_Proportion <- 1/(1+exp(-Pooled_logit)) #Back-transformation of logit transformation

*# Asymptotic 95% confidence interval of summary effect estimation with back-transformation of logit transformation [1]*

Meta_CI_95_inf <- round(1/(1+exp(-(Pooled_logit-1.96*sqrt(Meta_Variance)))), digits = 3)

Meta_CI_95_sup <- round(1/(1+exp(-(Pooled_logit+1.96*sqrt(Meta_Variance)))), digits = 3)

*# I^2 statistics [6]*

v <- ((nrow(File)-1)*sum(wi))/((sum(wi)^2)-sum(wi^2))

I2 <- round(tau2_PM/(tau2_PM+v), digits = 3)*100

*# 95% prediction interval [7]*

t_alpha <- c(12.706, 4.303, 3.182, 2.776, 2.571, 2.447, 2.365, 2.306, 2.262, 2.228) #First ten critical values of two-sided Student's t distribution, alpha = 0.05)

Transformed_Prediction_inf <- Pooled_logit-(t_alpha[nrow(File)-1]*sqrt(Meta_Variance+tau2_PM))

Transformed_Prediction_sup <- Pooled_logit+(t_alpha[nrow(File)-1]*sqrt(Meta_Variance+tau2_PM))

Prediction_95_inf <- round(1/(1+exp(-Transformed_Prediction_inf)), digits = 3) #Back-transformation of logit transformation

Prediction_95_sup <- round(1/(1+exp(-Transformed_Prediction_sup)), digits = 3) #Back-transformation of logit transformation

***###################################################################***

***########################## DISPLAY RESULTS #######################***

***##################################################################***

Meta_Proportion <- round(Meta_Proportion, digits = 3)

tau2_PM <- round(tau2_PM, digits = 4)

File <- select(File, -Logit, -Variance) #Remove irrelevant columns

File

str_glue("Summary effect estimate = {Meta_Proportion}")

str_glue("95% confidence interval = [{Meta_CI_95_inf}; {Meta_CI_95_sup}]")

str_glue("tau^2 = {tau2_PM}")

str_glue("I^2 = {I2} %")

str_glue("95% Prediction interval = [{Prediction_95_inf}; {Prediction_95_sup}]")

***###################################################################***

***############################ REFERENCES ##########################***

***###################################################################***

# [1] Schwarzer, G., Rücker, G. (2022). Meta-Analysis of Proportions. In: Evangelou, E., Veroniki, A.A. (eds) Meta-Research. Methods in Molecular Biology, vol 2345. Humana, New York, NY. https://doi.org/10.1007/978-1-0716-1566-9_10

# [2] Gart, J. J., & Zweifel, J. R. (1967). On the Bias of Various Estimators of the Logit and Its Variance with Application to Quantal Bioassay. Biometrika, 54(1/2), 181–187. https://doi.org/10.2307/2333861

# [3] Newcombe, R.G. (1998), Two-sided confidence intervals for the single proportion: comparison of seven methods. Statist. Med., 17: 857-872. https://doi.org/10.1002/(SICI)1097-0258(19980430)17:8<857::AID-SIM777>3.0.CO;2-E

# [4] van Aert, RCM, Jackson, D. Multistep estimators of the between-study variance: The relationship with the Paule-Mandel estimator. Statistics in Medicine. 2018; 37: 2616–2629. https://doi.org/10.1002/sim.7665

# [5] Borenstein, M., Hedges, L.V., Higgins, J.P.T. and Rothstein, H.R. (2010), A basic introduction to fixed-effect and random-effects models for meta-analysis. Res. Synth. Method, 1: 97-111. https://doi.org/10.1002/jrsm.12

# [6] Higgins, J.P.T. and Thompson, S.G. (2002), Quantifying heterogeneity in a meta-analysis. Statist. Med., 21: 1539-1558. https://doi.org/10.1002/sim.1186

# [7] IntHout J, Ioannidis JPA, Rovers MM, et al Plea for routinely presenting prediction intervals in meta-analysis BMJ Open 2016;6:e010247. doi: 10.1136/bmjopen-2015-010247
