# Supplementary material for: Perceptions of 3R implementation in European animal research: A systematic review, meta-analysis, and meta-synthesis of barriers and facilitators
Source: PLoS One. 2024 Mar 28;19(3):e0300031. doi: 10.1371/journal.pone.0300031 (PMC10977722; doi:10.1371/journal.pone.0300031)

S4 Appendix. Proportion meta-analysis plots for all analyzed survey items.

Q2: Existing 3R possibilities are currently optimally applied

I2 = 47.6%, τ 2 = 1.1438

Study	Proportion [95% CI]
**Bressers 2019 (N = 367)**	0.25 [0.21-0.3]
**Franco 2018 (N = 310)**	0.7 [0.65-0.75]
**van Luijk 2016 (N = 15)**	0.27 [0.11-0.52]
**Pooled effect (random)**	**0.409 [0.163-0.71]**
**Prediction interval**	**[0.003-0.993]**

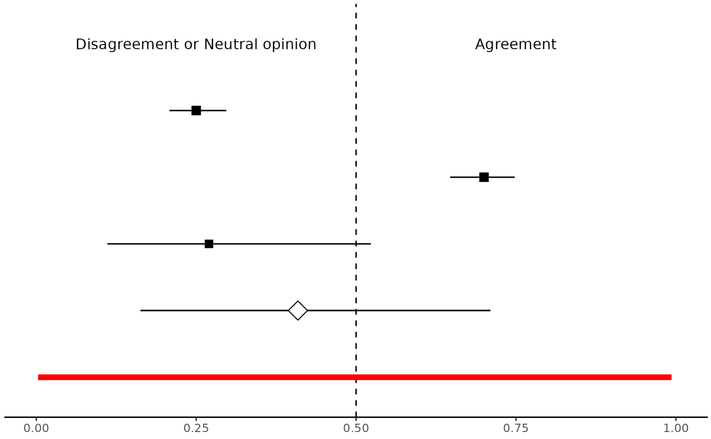

Q3: 3R implementation is important for animal welfare

I2 = 24%, τ 2 = 0.484

Study	Proportion [95% CI]
**van Luijk 2013 (N = 15)**	1 [0.8-1]
**Varoni 2023 (N = 501)**	0.92 [0.89-0.94]
**Pooled effect (random)**	**0.896 [0.705-0.969]**
**Prediction interval**	**[0-1]**

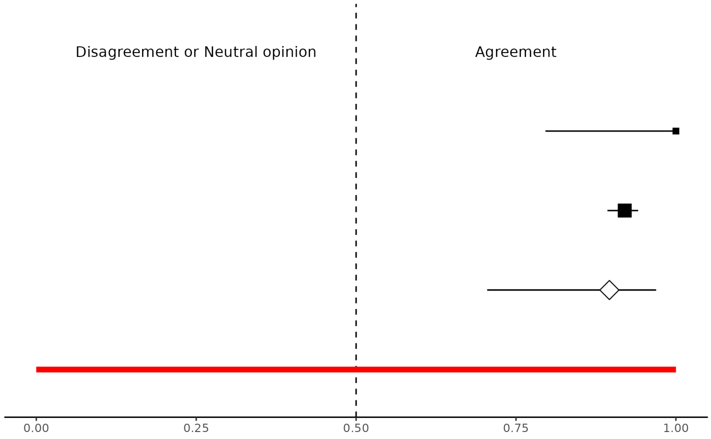

Q4: Refinement is the most important R

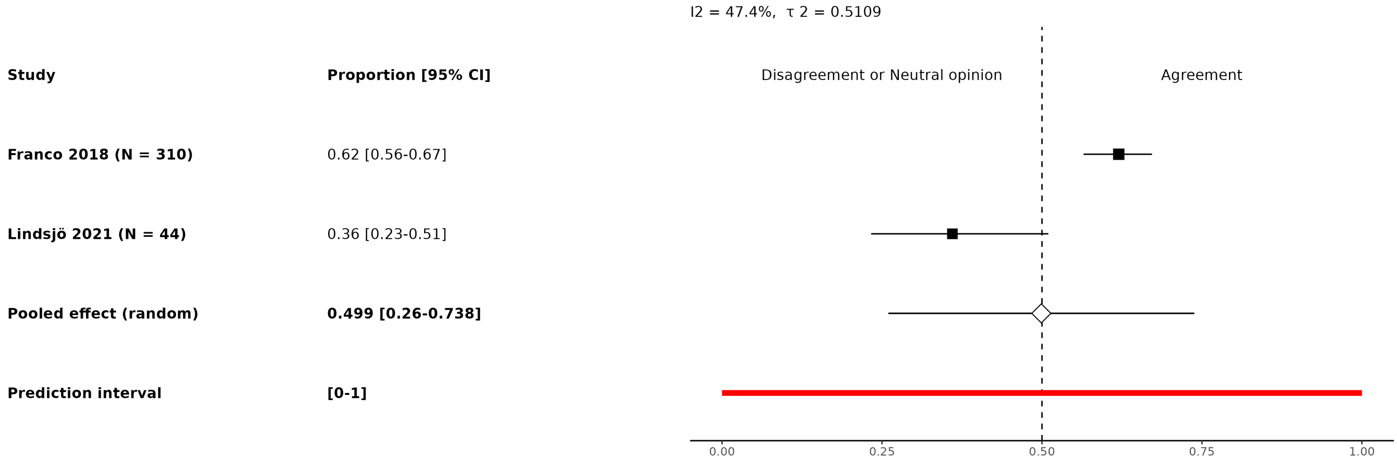

Q6*: 3R implementation leads to better quality of experimental results

*Lindsjö 2021 was excluded (41%, 95% CI 28-56%)

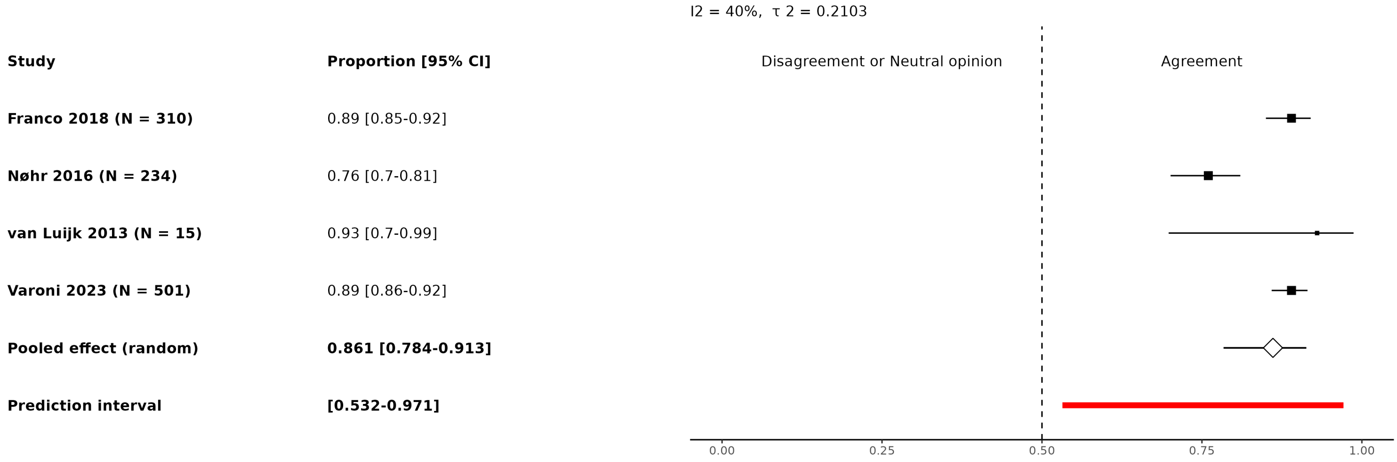

Q7*: Complete replacement will not be achieved in the foreseeable future

*Franco 2014 was excluded (99%, 95% CI 96-100%)

Study	Proportion [95% CI]
**Bressers 2019 (N = 367)**	0.71 [0.66-0.75]
**Franco 2018 (N = 310)**	0.67 [0.62-0.72]
**Lindsjö 2021 (N = 44)**	0.75 [0.6-0.86]
**Nøhr 2016 (N = 234)**	0.79 [0.73-0.84]
**Varoni 2023 (N = 501)**	0.68 [0.64-0.72]
**Pooled effect (random)**	**0.714 [0.666-0.757]**
**Prediction interval**	**[0.567-0.827]**

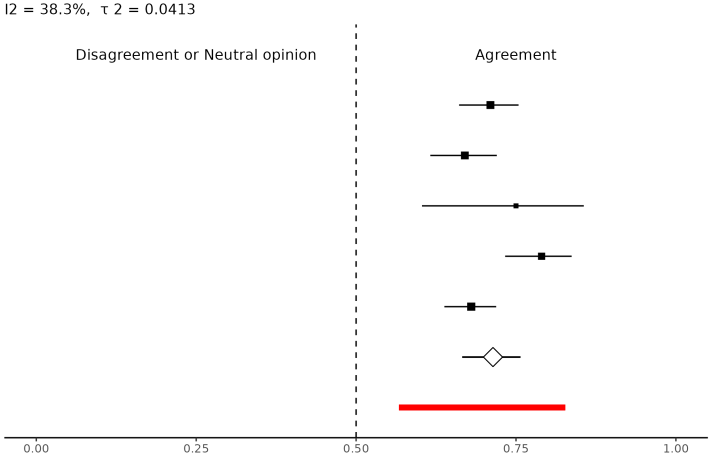

Q8: Replacement is possible for my field of study

Study	Proportion [95% CI]
**Crettaz von Roten 2018 (N = 510)**	0.25 [0.21-0.29]
**Franco 2014 (N = 206)**	0.12 [0.08-0.17]
**Franco 2018 (N = 310)**	0.24 [0.2-0.29]
**Nøhr 2016 (N = 234)**	0.18 [0.14-0.23]
**Varoni 2023 (N = 501)**	0.25 [0.21-0.29]
**Pooled effect (random)**	**0.207 [0.159-0.266]**
**Prediction interval**	**[0.083-0.429]**

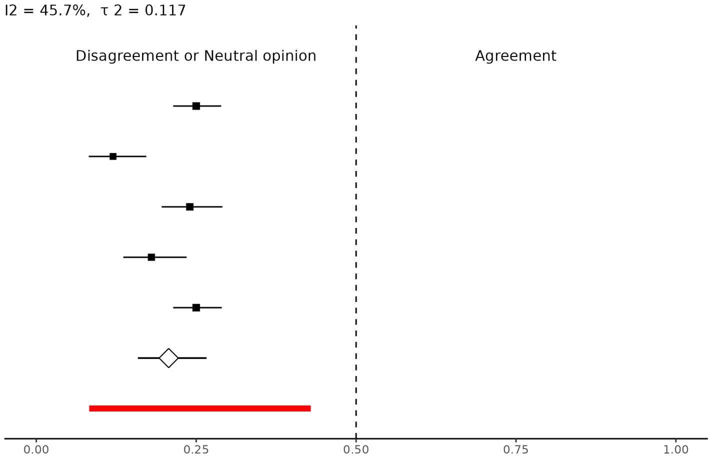

Q12: A dedicated budget is beneficial for 3R implementation

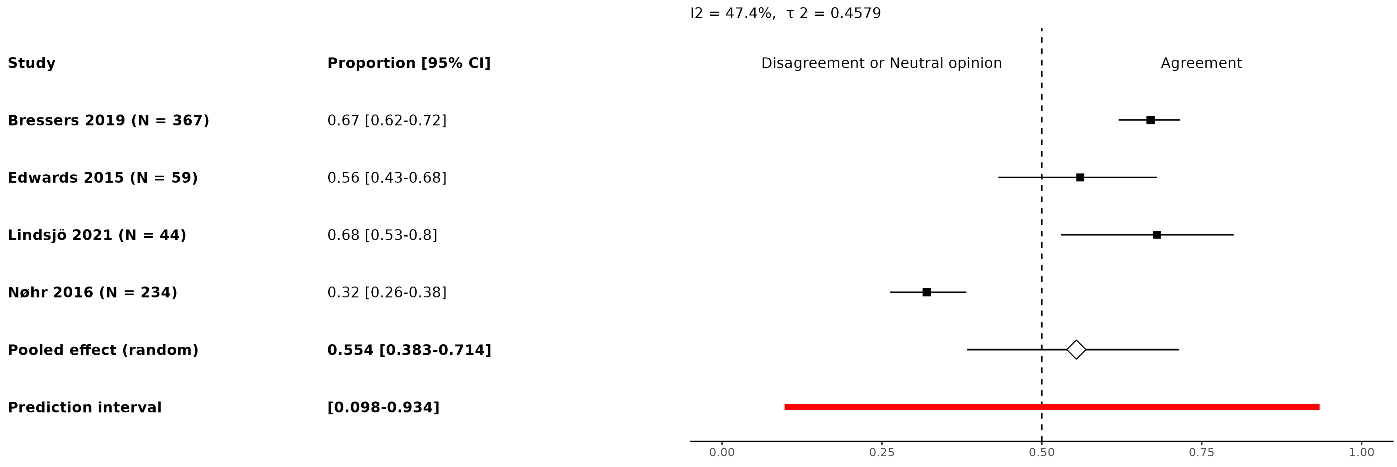

Q13: High standards from management/hierarchy is beneficial for 3R implementation

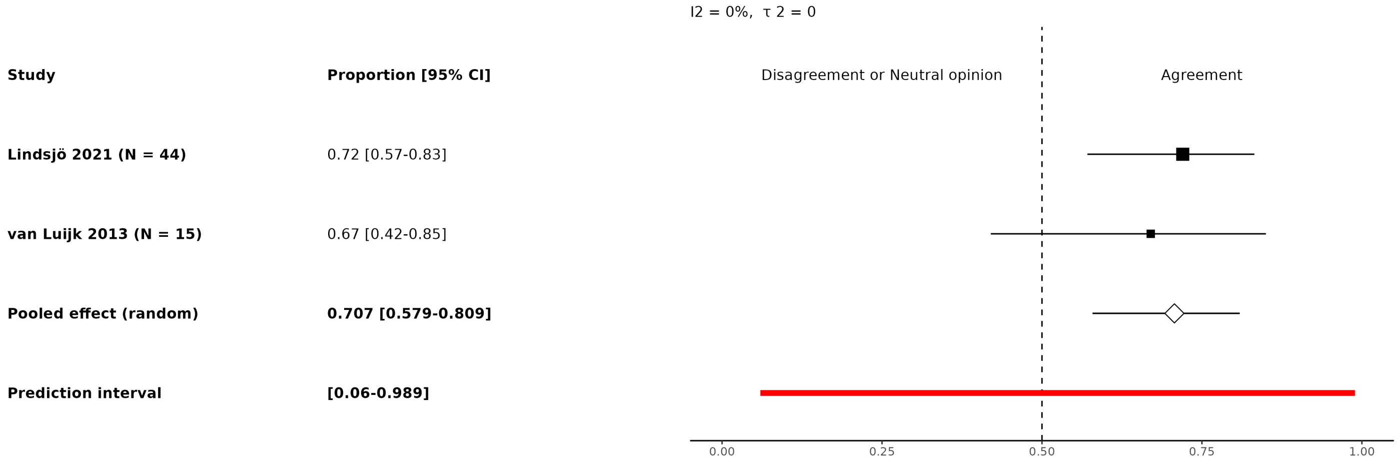

Q14: Improved regulation is beneficial for 3R implementation

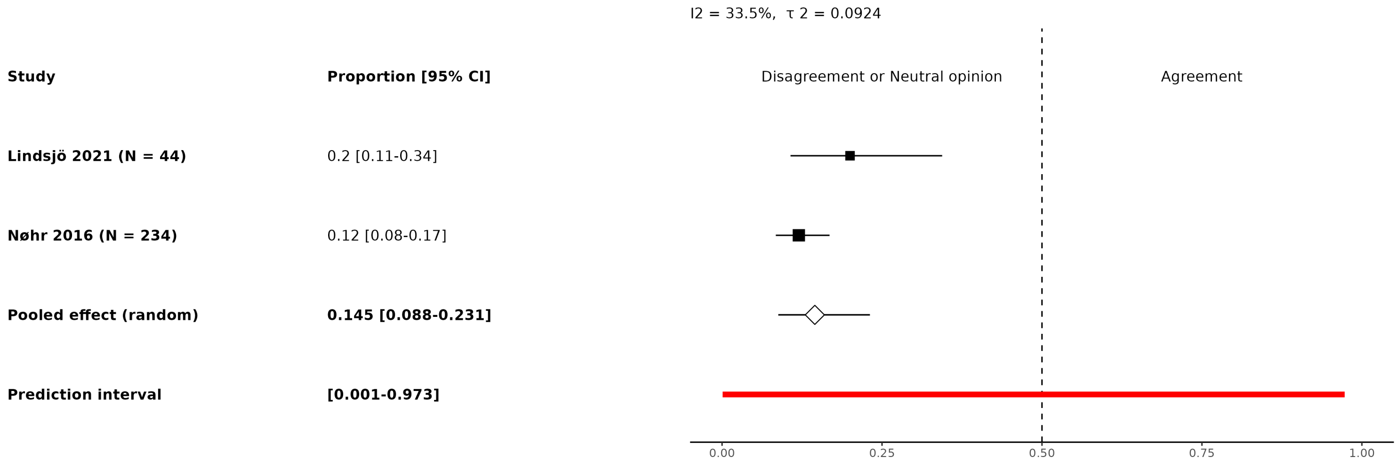

Q15: Improved work from Ethics Committees is beneficial for 3R implementation

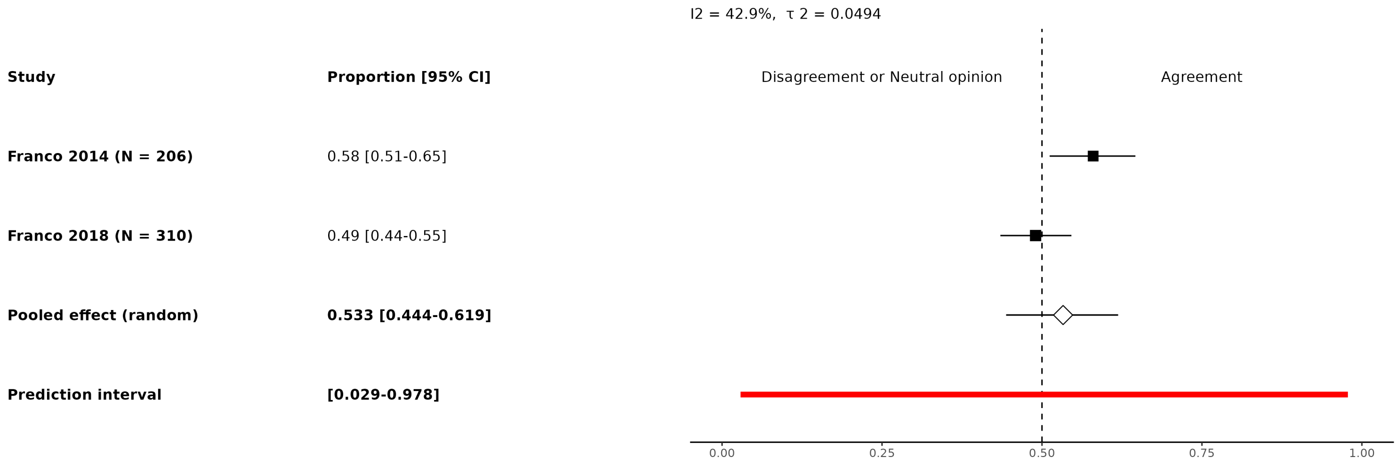

Q16: Dedicated animal specialists are beneficial to 3R implementation

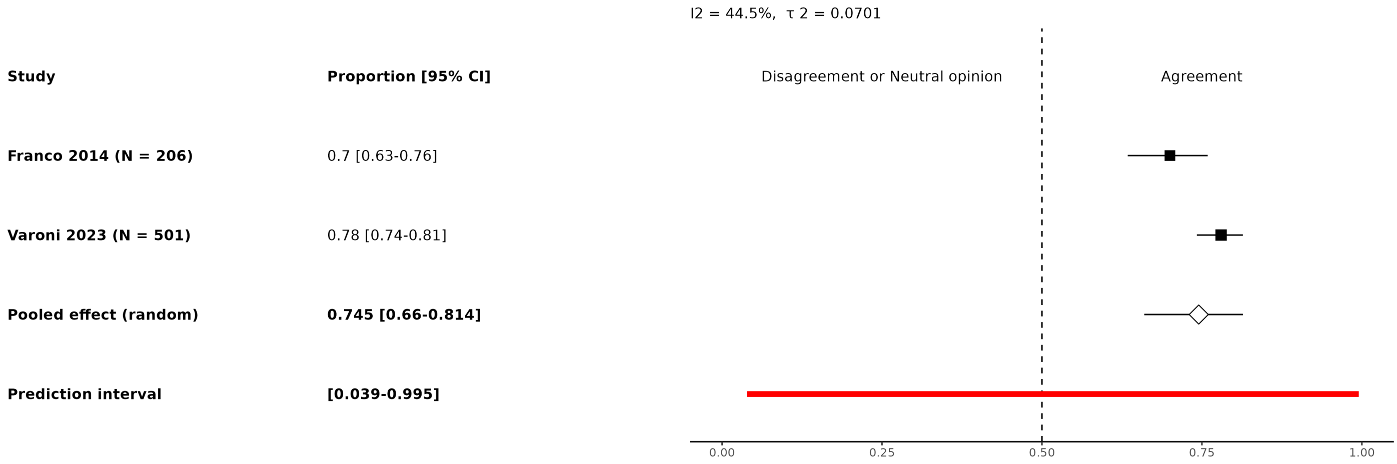

Q17: A dedicated literature search service is beneficial for 3R implementation

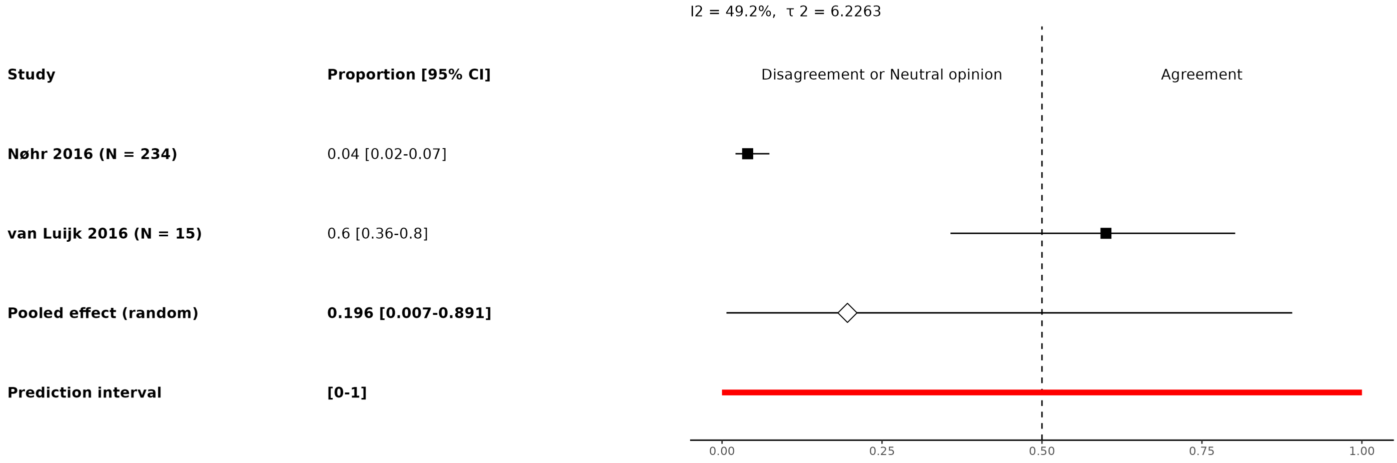

Q18: Education is beneficial for 3R implementation

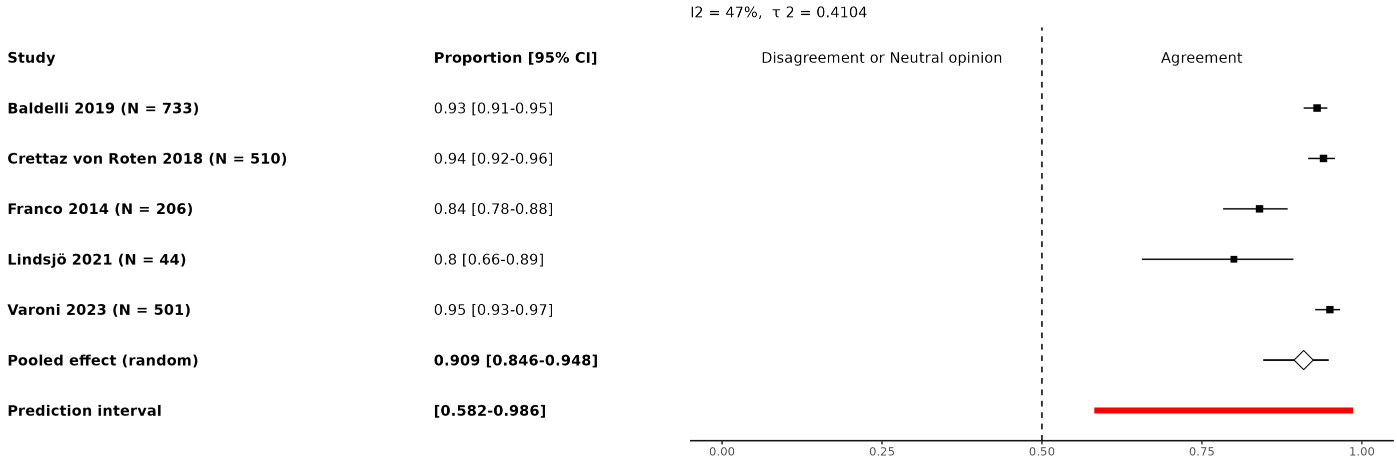

Q19: Knowledge exchange is beneficial for 3R implementation

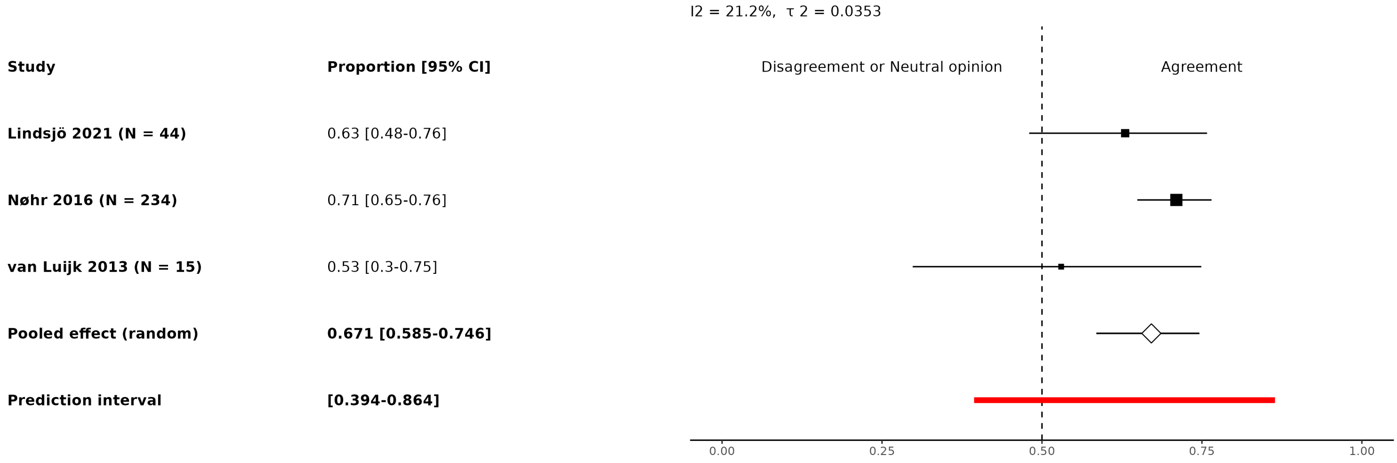

Q20: 3R implementation decreases appreciation by journals

Study	Proportion [95% CI]
**Bressers 2019 (N = 367)**	0.63 [0.58-0.68]
**Edwards 2015 (N = 59)**	0.31 [0.21-0.44]
**Franco 2018 (N = 310)**	0.54 [0.48-0.6]
**van Luijk 2016 (N = 15)**	0.27 [0.11-0.52]
**Pooled effect (random)**	**0.458 [0.295-0.631]**
**Prediction interval**	**[0.072-0.902]**

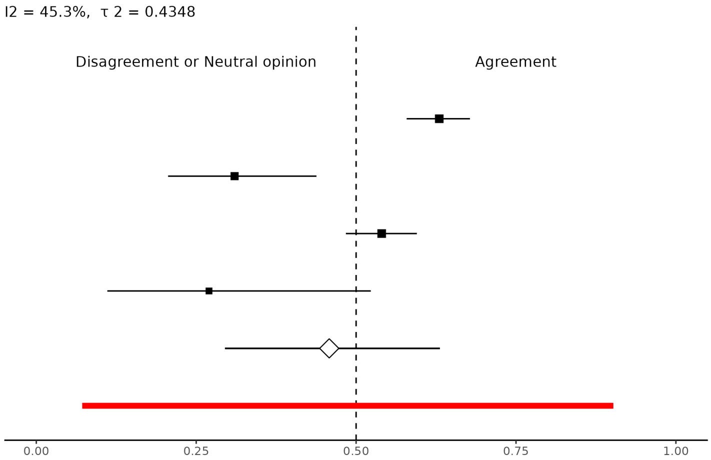

Q21*: 3R implementation increases research costs

*Bressers 2019 was excluded (68%, 95% CI 63-73%)

Study	Proportion [95% CI]
**Franco 2018 (N = 310)**	0.05 [0.03-0.08]
**Lindsjö 2021 (N = 44)**	0.2 [0.11-0.34]
**Nøhr 2016 (N = 234)**	0.07 [0.04-0.11]
**van Luijk 2013 (N = 15)**	0.13 [0.04-0.38]
**Pooled effect (random)**	**0.092 [0.048-0.17]**
**Prediction interval**	**[0.011-0.483]**

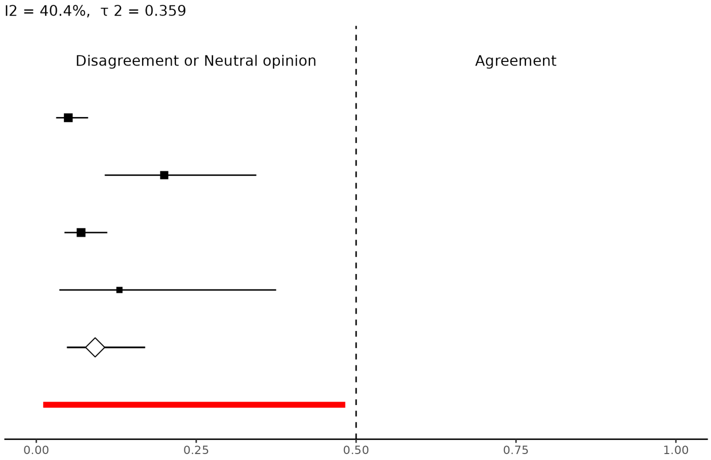

Q22: 3R implementation increases bureaucracy

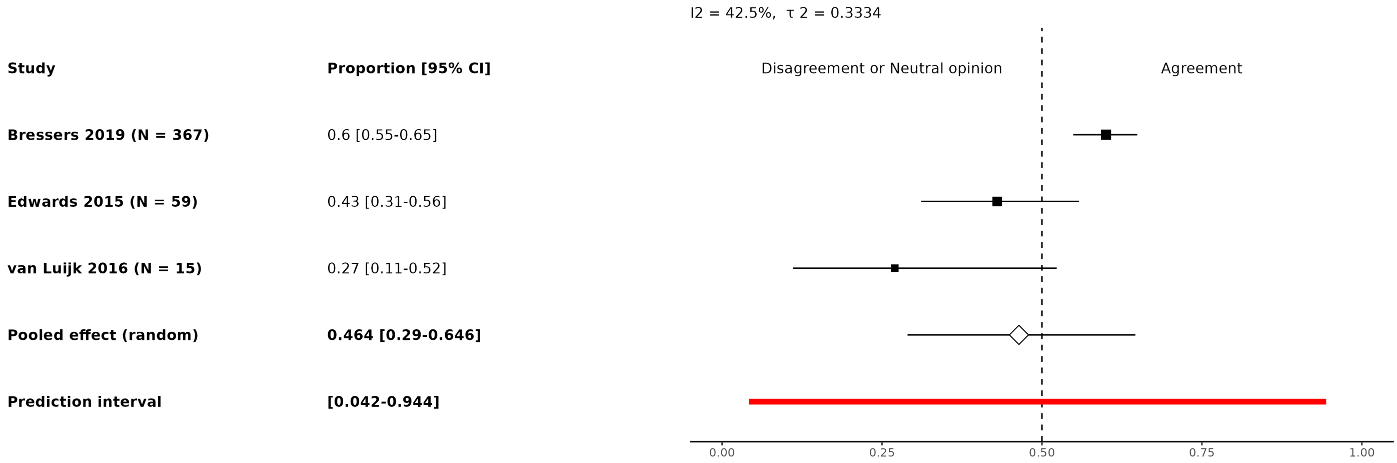

Q23*: 3R implementation slows down innovation

*Bressers 2019 was excluded (74%, 95% CI 69-78%)

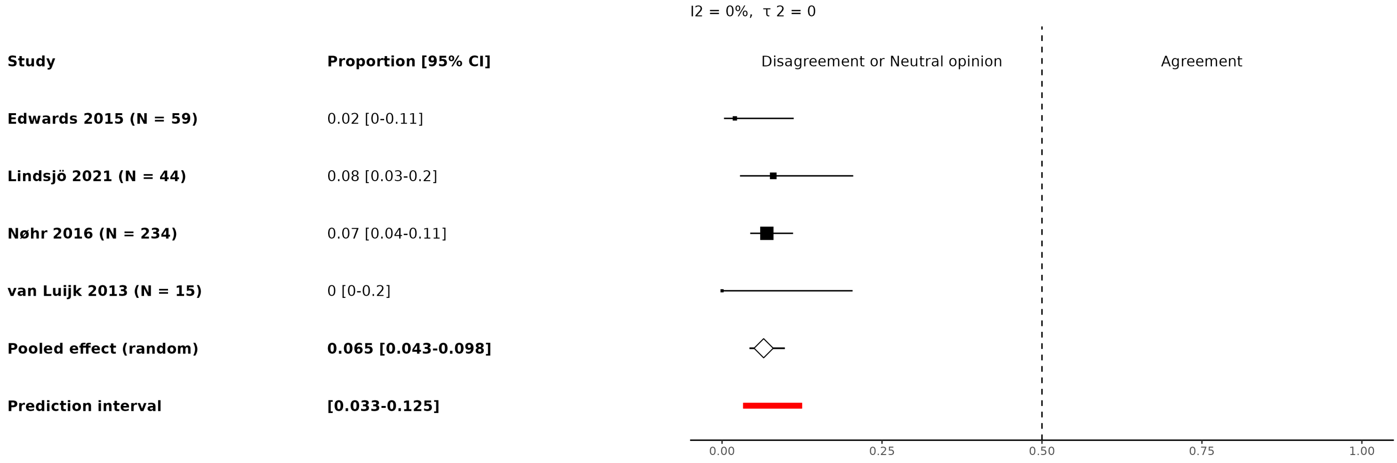

Q25*: 3R implementation leads to non-comparable data

*Bressers 2019 was excluded (89%, 95% CI 85-92%)

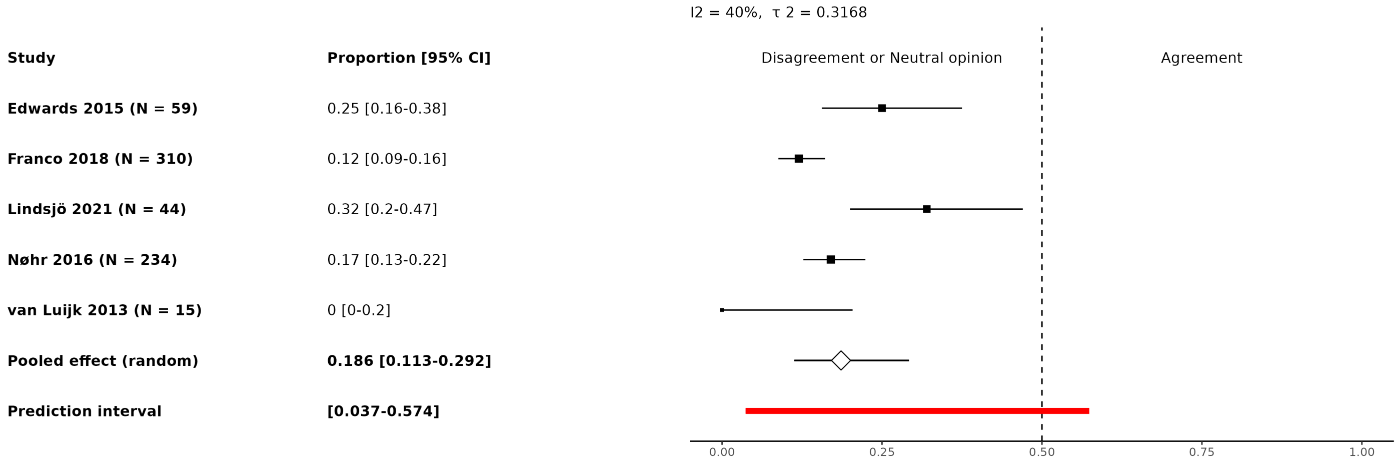

Supplement: S4 Appendix — (PDF) [file pone.0300031.s004.pdf]
