# Supplementary material for: Perceptions of 3R implementation in European animal research: A systematic review, meta-analysis, and meta-synthesis of barriers and facilitators
Source: PLoS One. 2024 Mar 28;19(3):e0300031. doi: 10.1371/journal.pone.0300031 (PMC10977722; doi:10.1371/journal.pone.0300031)
Supplement: Attachment — Submitted filename: Response to Reviewers.docx [file pone.0300031.s005.docx]

**Dear Dr. Ribeiro,**

We are grateful for your comments and the comments of the reviewer, which helped to improve the manuscript. Below we describe in detail how we responded to each comment.

**Academic editor:**

1. Please ensure that your manuscript meets PLOS ONE's style requirements, including those for file naming. The PLOS ONE style templates can be found at 
https://journals.plos.org/plosone/s/file?id=wjVg/PLOSOne_formatting_sample_main_body.pdf and 
https://journals.plos.org/plosone/s/file?id=ba62/PLOSOne_formatting_sample_title_authors_affiliations.pdf

RESPONSE: The manuscript has been edited to meet the formatting requirements of the templates.

2. Thank you for stating the following financial disclosure: "The authors acknowledge the support of the Swiss National Science Foundation (SNSF National Research Program (NRP) - 79 Advancing 3R – Animals, Research and Society, grant number 206432) in funding the APC." Please state what role the funders took in the study. If the funders had no role, please state: "The funders had no role in study design, data collection and analysis, decision to publish, or preparation of the manuscript." If this statement is not correct you must amend it as needed. 
Please include this amended Role of Funder statement in your cover letter; we will change the online submission form on your behalf.

RESONSE: The financial disclosure statement has been amended accordingly in the manuscript. This statement has been added to the cover letter as well.

3. We note that this manuscript is a systematic review or meta-analysis; our author guidelines therefore require that you use PRISMA guidance to help improve reporting quality of this type of study. Please upload copies of the completed PRISMA checklist as Supporting Information with a file name “PRISMA checklist”.

RESPONSE: The PRISMA checklist is reported in the Supporting Information section as “S1 Appendix”. The filename has been amended.

4. Please review your reference list to ensure that it is complete and correct. If you have cited papers that have been retracted, please include the rationale for doing so in the manuscript text, or remove these references and replace them with relevant current references. Any changes to the reference list should be mentioned in the rebuttal letter that accompanies your revised manuscript. If you need to cite a retracted article, indicate the article’s retracted status in the References list and also include a citation and full reference for the retraction notice.

RESPONSE: These references have been added to the reference list following Reviewer #1 suggestion:

Díez-Solinska A, Vegas O, Azkona G. Refinement in the European Union: A Systematic Review. Animals. 2022;12: 3263. doi:10.3390/ani12233263

Goñi-Balentziaga O, Vila S, Ortega-Saez I, Vegas O, Azkona G. Professional Quality of Life in Research Involving Laboratory Animals. Animals. 2021;11: 2639. doi: 10.3390/ani11092639

Goñi-Balentziaga O, Ortega-Saez I, Vila S, Azkona G. Working with laboratory rodents in Spain: a survey on welfare and wellbeing. Lab Anim Res. 2021;37. doi: 10.1186/s42826-021-00098-w

Goñi-Balentziaga O, Azkona G. Perceived professional quality of life and mental well-being among animal facility personnel in Spain. Laboratory Animals. 2023;0. doi:[10.1177/00236772231187177](https://doi.org/10.1177/00236772231187177)

**Reviewer #1:**

Dear Authors,
Congratulations on your work. I think it is very well designed, analysed and written.

RESPONSE: Thank you very much for this positive feedback.

However, being a work oriented to the European Union, I am missing the work of a Spanish research group that has been working in this field in the last few years and that could be mentioned, since American groups are mentioned, it would not be superfluous to mention European groups. The works would be:
- PMID: 34315536 PMCID: PMC8314439 DOI: 10.1186/s42826-021-00098-w
- PMID: 34573605 PMCID: PMC8465412 DOI: 10.3390/ani11092639
- PMID: 35655241 PMCID: PMC9161537 DOI: 10.1186/s42826-022-00124-5
- PMID: 36496784 PMCID: PMC9735736 DOI: 10.3390/ani12233263
- PMID: 37684027 DOI: 10.1177/00236772231187177
This is just a recommendation, which I think would be interesting to be discussed in the discussion.
RESPONSE: Thank you for these suggestions. One of these references appeared in our systematic search, but could not be included in our analysis as it was not meeting all inclusion criteria. As the conclusions of some of these references relate to some aspects of our discussion, we included them where appropriate.
